# Muscle architecture dynamics modulate performance of the superficial anterior temporalis muscle during chewing in capuchins

**DOI:** 10.1038/s41598-020-63376-y

**Published:** 2020-04-14

**Authors:** Myra F. Laird, Michael C. Granatosky, Andrea B. Taylor, Callum F. Ross

**Affiliations:** 1 Department of Integrative Anatomical Sciences, University of Southern California, Los Angeles, CA USA; 2 Department of Anatomy, New York Institute of Technology, Old Westbury, NY USA; 3 Basic Science Department, Touro University, Vallejo, CA USA; 4 Department of Organismal Biology and Anatomy, University of Chicago, Chicago, IL USA

**Keywords:** Mandibular muscles, Biological anthropology

## Abstract

Jaw-muscle architecture is a key determinant of jaw movements and bite force. While static length-force and force-velocity relationships are well documented in mammals, architecture dynamics of the chewing muscles and their impact on muscle performance are largely unknown. We provide novel data on how fiber architecture of the superficial anterior temporalis (SAT) varies dynamically during naturalistic feeding in tufted capuchins (*Sapajus apella*). We collected data on architecture dynamics (changes in muscle shape or the architectural gear ratio) during the gape cycle while subjects fed on foods of different mechanical properties. Architecture of the SAT varied with phases of the gape cycle, but gape distance accounted for the majority of dynamic changes in architecture. In addition, lower gear ratios (low muscle velocity relative to fascicle velocity) were observed when animals chewed on more mechanically resistant foods. At lower gear ratios, fibers rotated less during shortening resulting in smaller pinnation angles, a configuration that favors increased force production. Our results suggest that architectural dynamics may influence jaw-muscle performance by enabling the production of higher bite forces during the occlusal phase of the gape cycle and while processing mechanically challenging foods.

## Introduction

The capacity of mammal jaw-elevator muscles to generate force and transmit it to the bite point is limited by two fundamental and inter-related constraints^1^: i) the length-force and force-velocity properties of skeletal muscle; and ii) the third-class lever arrangement of the jaw-elevator muscles^2–4^. In combination with the geometry of the feeding system (i.e., height of the jaw joint above the tooth row and bite location along the tooth row)^5,6^ these fundamental constraints impose trade-offs between bite force, jaw gape (vertical displacement of the lower jaw), and bite point (bite location along the toothrow) that must be confronted by natural selection.

While these static aspects of mammal feeding-system design are well studied with respect to bite force and jaw movement, e.g. refs. ^2–7^, the impacts of feeding-system dynamics—jaw and muscle kinematics—are less well understood. Dynamic changes in jaw-elevator muscle lengths during chewing in three species of primates have been estimated from three-dimensional (3D) jaw kinematic data^8^, but these data cannot be transformed into estimates of muscle and bite force kinetics without detailed information on static and dynamic properties of muscle architecture.

Here we examine how fiber architecture of the superficial anterior temporalis (SAT) muscle varies dynamically during feeding in tufted capuchins (*Sapajus apella*). Tufted capuchin feeding systems are of interest because of their ability to exploit mechanically challenging foods at relatively large jaw gapes^9–11^. Compared with untufted capuchins, tufted capuchins have a suite of craniodental and muscular features well-suited for the production of high bite forces, including: a thicker and deeper mandible; more robust temporal bone; larger incisors, canines, and molars; thicker enamel of the postcanine dentition; and relatively greater physiological cross section areas (PCSAs) of the superficial masseter and temporalis muscles^10–12^. Tufted capuchins also have features that facilitate the production of large muscle and bite forces without compromising gape; specifically, increased jaw-elevator PCSAs through added muscle mass rather than shorter fiber lengths and larger pinnation angles^12^. Thus, tufted capuchins are an excellent model species to investigate how dynamic muscle architecture varies with gape and muscle performance.

### Skeletal gearing and the gape cycle

Muscle architecture can be defined as the internal arrangement of fibers relative to the force-generating axis of a muscle, e.g. ref. ^13^. The architectural arrangement of the jaw-elevator muscles impacts the capacity of these muscles to generate their maximum force across changing muscle lengths during jaw opening and closing. The maximum force-generating capacity of a muscle has been shown to be proportional to its PCSA^14^, which is a function of its mass, fiber lengths, and pinnation angles^15^. However, there is a trade-off between force and gape such that the jaw-elevator muscles produce the greatest amount of bite force when the jaw is at or near an optimal gape, and bite force decreases at smaller and (especially) larger gapes^2,7,16–19^; but see ref. ^20^.

**Figure 1:**
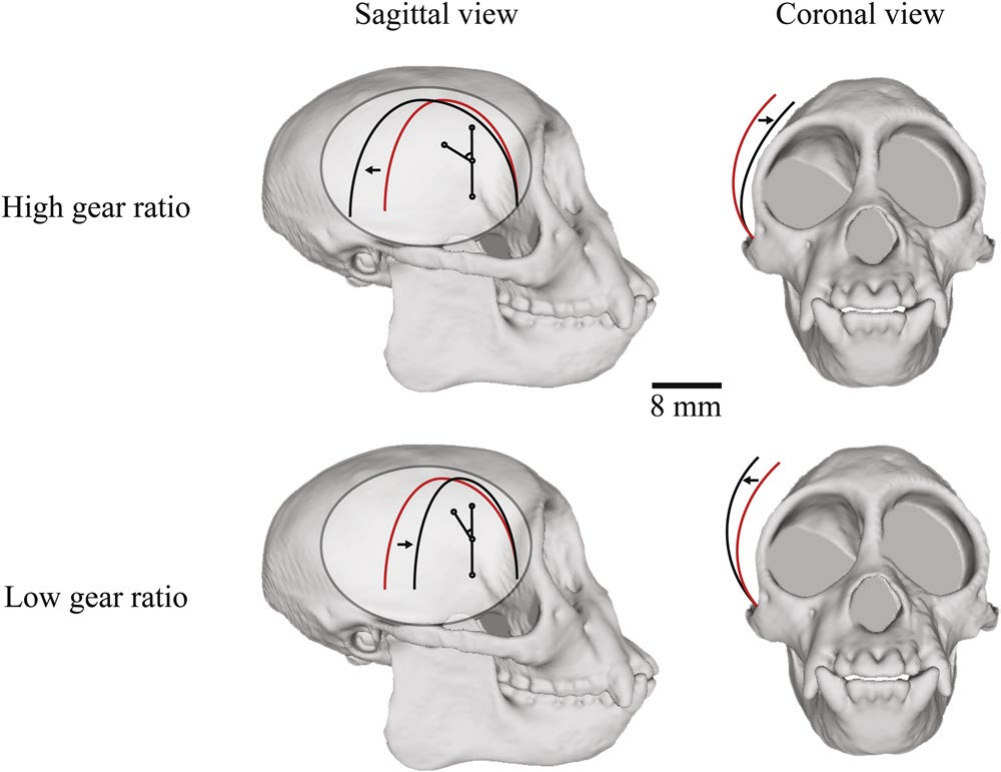
Schematic of changes in architectural gear ratio (AGR) and muscle bulging of the temporalis in sagittal and coronal planes from scaled CT images. The red lines represent the original position of the muscle and fascicle before shortening. At high gear ratios, the increase in pinnation during shortening results in increased muscle anteroposterior thickness (anteroposterior expansion shown in sagittal view, black arrows). At low gear ratios, pinnation angle decreases and muscle mediolateral width increases (mediolateral expansion shown in coronal view, black arrows). The shaded ellipse shown in sagittal view represents the tendinous sheath.

The jaw adductor muscles can potentially bypass force-gape constraints through dynamic changes in shape (Fig. 1), described as a muscle’s architectural gear ratio (AGR). The AGR has been defined as the ratio of whole-muscle strain/fascicle strain^21,22^ or its temporal derivative, whole-muscle velocity/fascicle velocity^23–26^. At lower gear ratios—low muscle strain or shortening relative to fascicle strain or shortening—muscle fibers rotate less during shortening, resulting in a smaller pinnation angle and favoring increased force production. In contrast, at high gear ratios greater fiber rotation and a large pinnation angle result in high-velocity muscle shortening relative to fiber shortening, a configuration hypothesized to favor muscle shortening rather than force production. Importantly, changes in AGR have been demonstrated in isolated muscle preparations indicating that muscle shape changes are not mediated by the central nervous system^24^. This finding suggests force or velocity benefits from AGR can be independent of muscle activation.

The four phases of the gape cycle^27^ vary in jaw acceleration and in muscle activity relating to bite force production in ways that might be expected to affect whole fascicle velocity, muscle velocity and the AGR. Starting at maximum gape, jaw closing begins with a fast close (FC) phase, which continues until jaw elevation is slowed by the teeth encountering the food—the start of slow close (SC). Activity of the jaw-elevator muscles rapidly increases during SC until minimum gape is achieved. Minimum gape is followed by the slow opening (SO) phase, when the jaw-elevator muscles are de-activated, and the jaw depresses slowly as the tongue protrudes to engage with the food bolus. Slow opening ends and transitions to fast opening (FO) when the jaw is quickly depressed until the time of maximum gape^1^. Importantly, gearing within a given muscle can vary from one contraction to another, depending on a number of factors. For example, in the gastrocnemius muscle, the AGR decreases as muscle force increases^23–26^, suggesting that muscle gearing might function as a passive mechanism for modulating force output. This raises the possibility that passive variability in AGR between gape cycles might contribute to inter-cycle muscle performance as food bolus properties change during a feeding sequence.

Static muscle architecture of the jaw-elevator muscles is well documented in non-primate mammals^19,28,29^ and strepsirrhine^30^ and anthropoid^12,31–33^ primates, including humans^34^. Likewise, the impact of sarcomere-length changes from minimum gape (jaws closed) to maximum gape (jaws open) on fiber length and PCSA has been documented in a handful of small mammals^35^ and in several macaque species^36^. In contrast, little is known about how mammal jaw-elevator muscle architecture changes dynamically or how these changes impact muscle performance during feeding. Whole muscle lengths have been shown to change non-linearly and in muscle-specific fashion over the gape cycle^8^, impacting a muscle’s force-production capacity through its length-force and force-velocity properties. However, the dynamic changes in muscle pinnation angles that produce complex relationships between the shortening strain and velocity of whole muscles, and those of muscle fibers and fascicles, are virtually unstudied in mammal feeding systems. Notably, the literature is dominated by *in situ* studies of locomotor muscles in non-primate mammals, often under conditions of maximal muscle activation, and by *in vivo* studies of limb muscles in humans during bicycling^23–26,37,38^. In contrast, there is a distinct lack of studies of muscle architecture dynamics during natural behaviors and at sub-maximal muscle activation levels^22,39^ and this is crucial because the presence of inactive or passive muscle fibers can affect the way that active fibers rotate and shorten during contraction, in turn impacting force production.

The primate feeding system is ideal for studies of how muscle architecture dynamics influence muscle performance during natural behaviors because a range of gapes at submaximal muscle-recruitment can be evoked by varying food geometric and material properties^30,40–43^. Here, muscle performance is defined as the ability to generate forces necessary for food break down. We take advantage of the fact that bite force is only applied during a very specific phase of the mammalian gape cycle^44–46^ to link dynamic changes in architecture with bite force, and the fact that jaw-muscle and bite force vary with food mechanical properties, with relatively higher muscle and bite forces associated with more mechanically challenging foods^5,41^. Using bi-planar videoradiography and the X-ray Reconstruction of Moving Morphology (XROMM) workflow, dynamic muscle architecture data can be collected while animals feed unrestrained.

**Figure 2:**
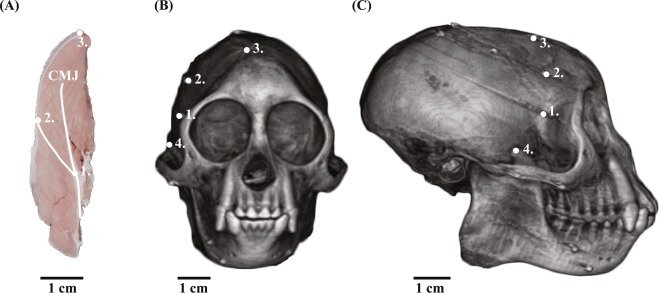
(**A**) Anterior view of a coronal section of a capuchin right temporalis. In this study, tantalum beads were sutured at the ends of a single fascicle at its central myotendinous junction (CMJ; 1) and its superficial termination at the temporal fascia (2). A third marker was placed at the temporal fascia line roughly in the same coronal plane as the fascicle (3). The SAT fascicles are oblique to coronal and sagittal planes (cf. Fig. 1). **(B)** Coronal view from a CT scan of a subject animal showing the positions of each of the muscle beads (1–3) and the positioning of the tip of the coronoid process (4), which was used to measure whole-muscle length. **(C)** Sagittal view of a CT scan of a subject animal showing the positioning of the muscle markers (1–3) and the positioning of the tip of the coronoid process (4).

The temporalis muscle is an attractive focus for initial work on jaw- muscle architecture dynamics because its importance for generating vertical bite forces is well documented in primates^41,47^ and it is readily accessible for marker placement. Bipennate in coronal section, the temporalis takes origin from the rigid bone of the calvaria medially and the deep surface of the more compliant temporal fascia laterally and converges on a central tendon that attaches to the superior and anterior coronoid process and ramus of the mandible (Fig. 2A). The mechanical properties of the temporal fascia are important for the mechanics of the zygomatic arch, one of the most highly strained areas in the primate skull, making the *in vivo* function of this region of comparative and evolutionary significance^48^.

#### Hypotheses to be tested

We focused on changes in muscle architectural variables during *chewing* gape cycles and evaluated the relative importance of gape distance on architectural dynamics. We also investigated changes in dynamic muscle architecture in relation to food mechanical properties (FMPs). We tested the following three hypotheses:

Hypothesis 1: Muscle architecture of the SAT varies dynamically with gape distance within gape cycles. Depression and elevation of the jaw during chewing is expected to produce cyclic changes in muscle length, fascicle length, and fascicle orientation in coronal and sagittal planes. Thus, we predicted that i) fascicle lengths would be the shortest, coronal and sagittal fascicle angles would be largest, and AGRs would be lowest, at and approaching minimum gape; and ii) sagittal angle would vary more than coronal angle during the gape cycle because the coronoid process and central tendon of the muscle—to which the fascicles attaches distally—displace more in sagittal than coronal planes during chewing^8^.

Hypothesis 2: After controlling for gape, fascicle velocity, muscle velocity and AGR vary across gape-cycle phases. The FC to SC transition is the time in the gape cycle when the teeth engage the food, bite forces are first produced, and jaw elevation slows^27^. Thus, we predicted that across all food mechanical properties (FMPs), fascicle and muscle velocities would be higher and AGRs would be lower at the FC to SC transition compared to other gape cycle phases, after controlling for gape. While the jaw-elevator muscles are not active during jaw opening, we predict higher fascicle and muscle velocities and lower AGRs compared to jaw closing, reflecting changes in jaw acceleration. We base these predictions regarding dynamic changes *within* chewing gape cycles on previous work comparing *between* limb movement cycles at different torques^38^. Specifically, during concentric activation of human gastrocnemius while cycling, variation in AGR was found to be driven primarily by differences in the *forces* generated by the muscle, not in muscle shortening *velocity*. As force increased, muscle bulging and fascicle rotation decreased, fascicle shortening velocity increased, and AGR decreased^38^.

Hypothesis 3: SAT AGR varies with FMPs. Lower AGRs are associated with low muscle velocity relative to fiber velocity and with better force production in both isometric^24^ and dynamic contexts^38^. Thus, we predict that phases of the gape cycle relating to force production or changes in jaw acceleration for more mechanically challenging foods would be characterized by lower AGRs compared to less challenging foods.

## Results

### Hypothesis 1: Muscle architecture of the SAT varies dynamically with gape distance within gape cycles

**Figure 3:**
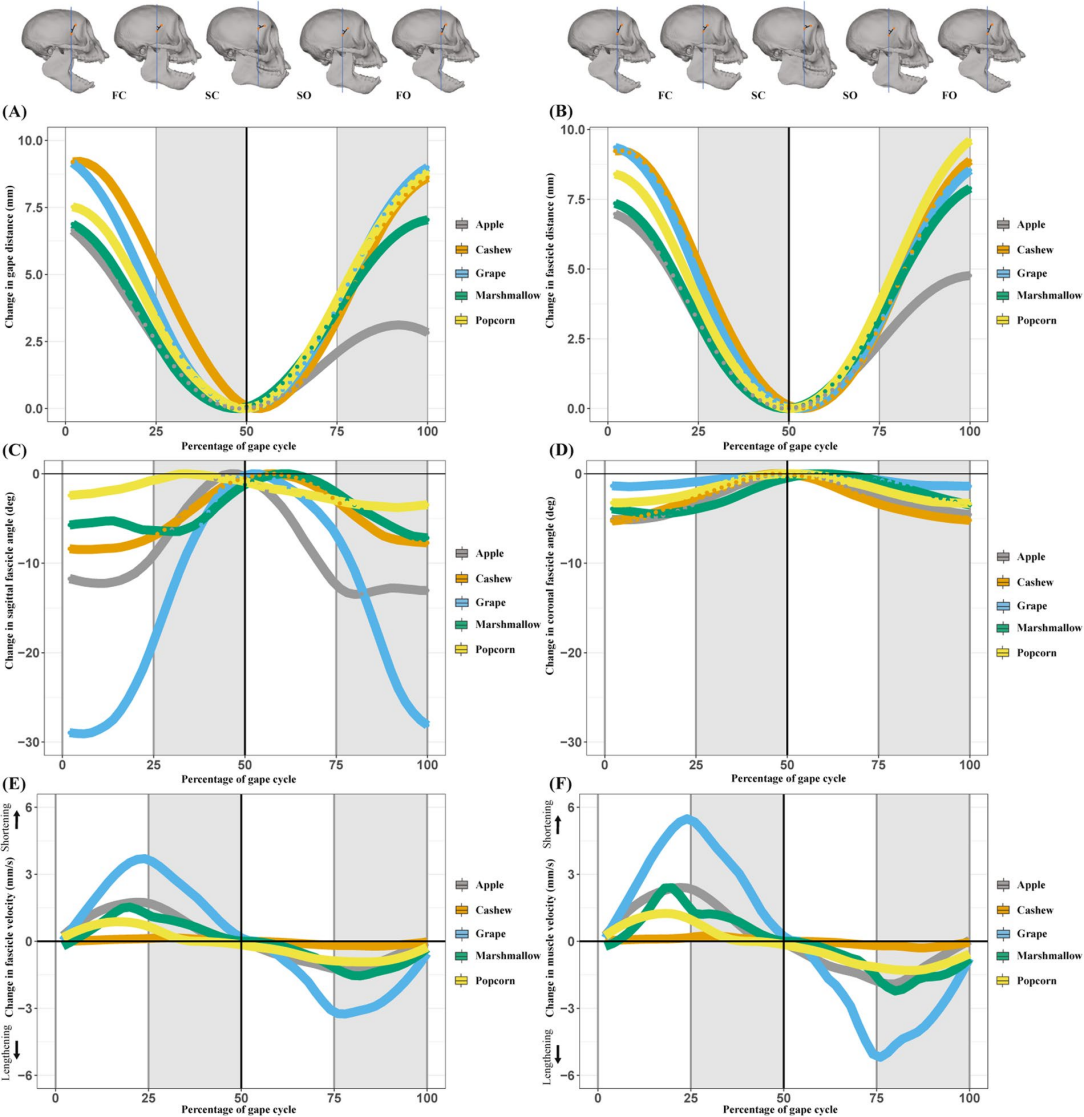
Averages of 30-33 gape cycles (93 total) from the three subject animals. **(A)** Gape distance and **(B)** fascicle distance, over the standardized gape cycle: maximum gape occured at the FO/FC transition, minimum gape at the SC/SO transition. Sagittal **(C)** and coronal **(D)** fascicle angles changed over the gape cycle. The largest sagittal and coronal fascicle angles occured at minimum gape; the smallest occured at maximum gape. Sagittal fascicle angle changed approximately twice as much as coronal fascicle angle over the gape cycle. The timing of minimum sagittal and coronal fascicle angles did not signficantly differ between food types. **(E)** LOESS fit (with a 25% smoothing span) of fascicle length velocity during the gape cycle. Fascicles shortened to the SC/SO transition (minimum gape), with the maximum shortening velocity around the FC/SC transition and maximum lengthening velocity around the SO/FO transition. **(F)** LOESS fit (with a 25% smoothing span) of muscle length velocity during the gape cycle. Maximum shortening velocity occurred around the FC/SC, minimum lengthening velocity occurred around the SO/FO transition. Figure generated in R (2017; https://www.R-project.org).

**Figure 4:**
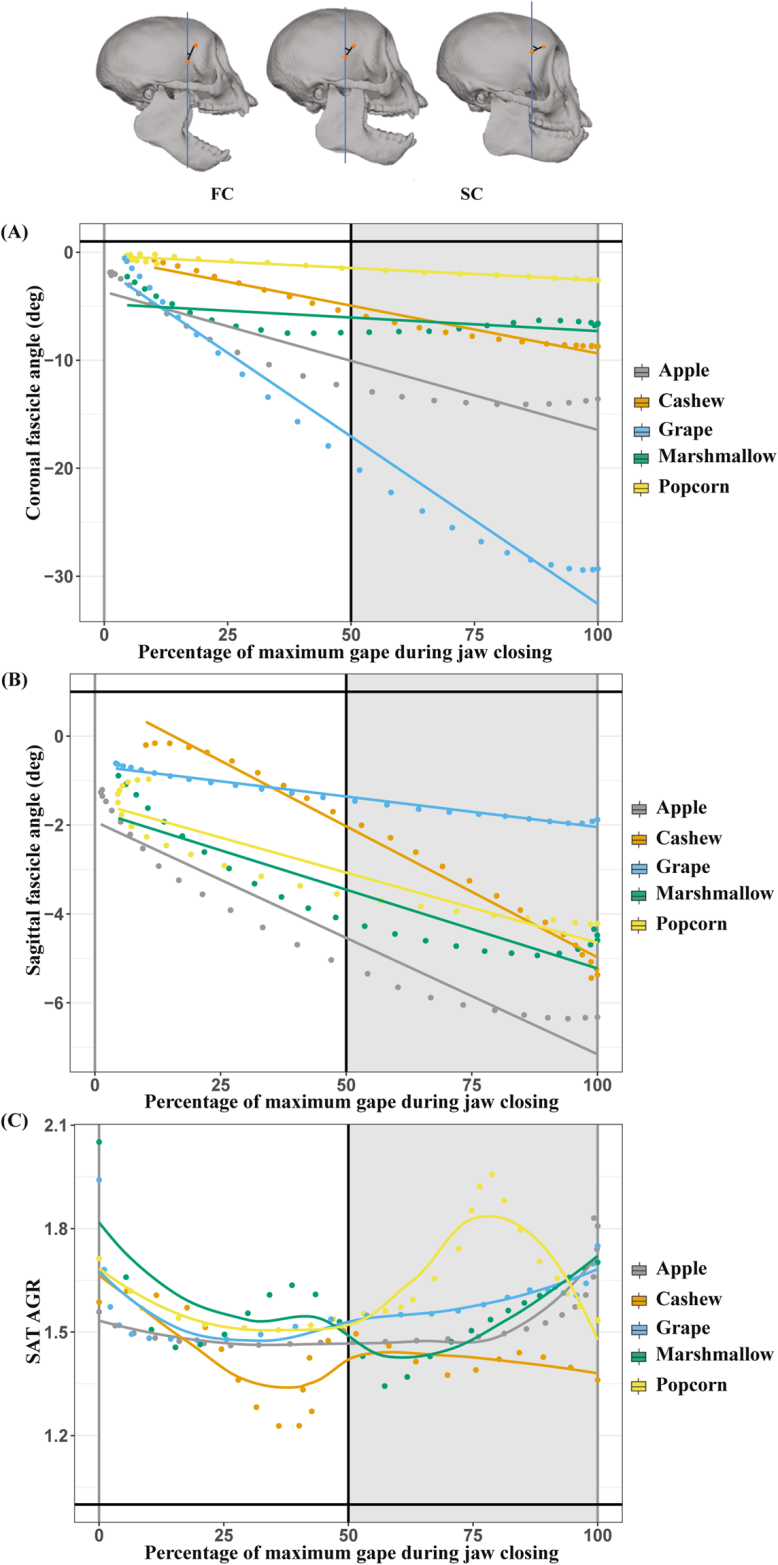
(**A**) Coronal and **(B)** sagittal fascicle angles decreased with gape distance. The slopes of these relationships varied with food mechanical properties. (**C**) The superficial anterior temporalis architectural gear ratio (SAT AGR) decreased with normalized gape distance until about halfway to maximum gape, i.e., the FC/SC transition. Following the halfway point, SAT AGR increased. Figure generated in R (2017; https://www.R-project.org).

As predicted, fascicle and whole muscle lengths were smallest at minimum gape (jaw closed) and largest at maximum gape (jaw open) (Fig. 3A,B). Fascicle and muscle lengths were strongly positively correlated with gape distance during both jaw opening and closing such that, as gape distance increased (the jaw depressed), fascicle and muscle length increased (fascicle: *p* < 0.01, R^2^m = 0.82, R^2^c = 0.91; muscle: *p* < 0.01, R^2^m = 0.99, R^2^c = 0.99). Likewise, coronal and sagittal fascicle angles were largest at minimum gape and smallest at maximum gape (Fig. 3C,D) and sagittal fascicle angles exhibited at least twice as much variation compared to coronal fascicle angles across the gape cycle. Sagittal and coronal fascicle angles were negatively correlated with gape distance during jaw opening and closing such that fascicle angles decreased significantly as gape increased (sagittal: *p* < 0.01, R^2^m = 0.33, R^2^c = 0.69; coronal: *p* < 0.01, R^2^m = 0.46, R^2^c = 0.72) (Fig. 4A,B).

Instantaneous fascicle and muscle shortening (0–50% of the gape cycle) velocities increased from maximum gape until about 25% of the gape cycle when they reached their maximum shortening velocity (Fig. 3E,F). Both fascicle and muscle shortening velocities then decreased until at or near minimum gape, when they became lengthening velocities (50–100% of the gape cycle). Maximum lengthening velocities occurred at ~75% of the gape cycle and then decreased during the final 25% of the gape cycle. The range of variation was slightly larger for muscle compared to fascicle velocities.

**Figure 5:**
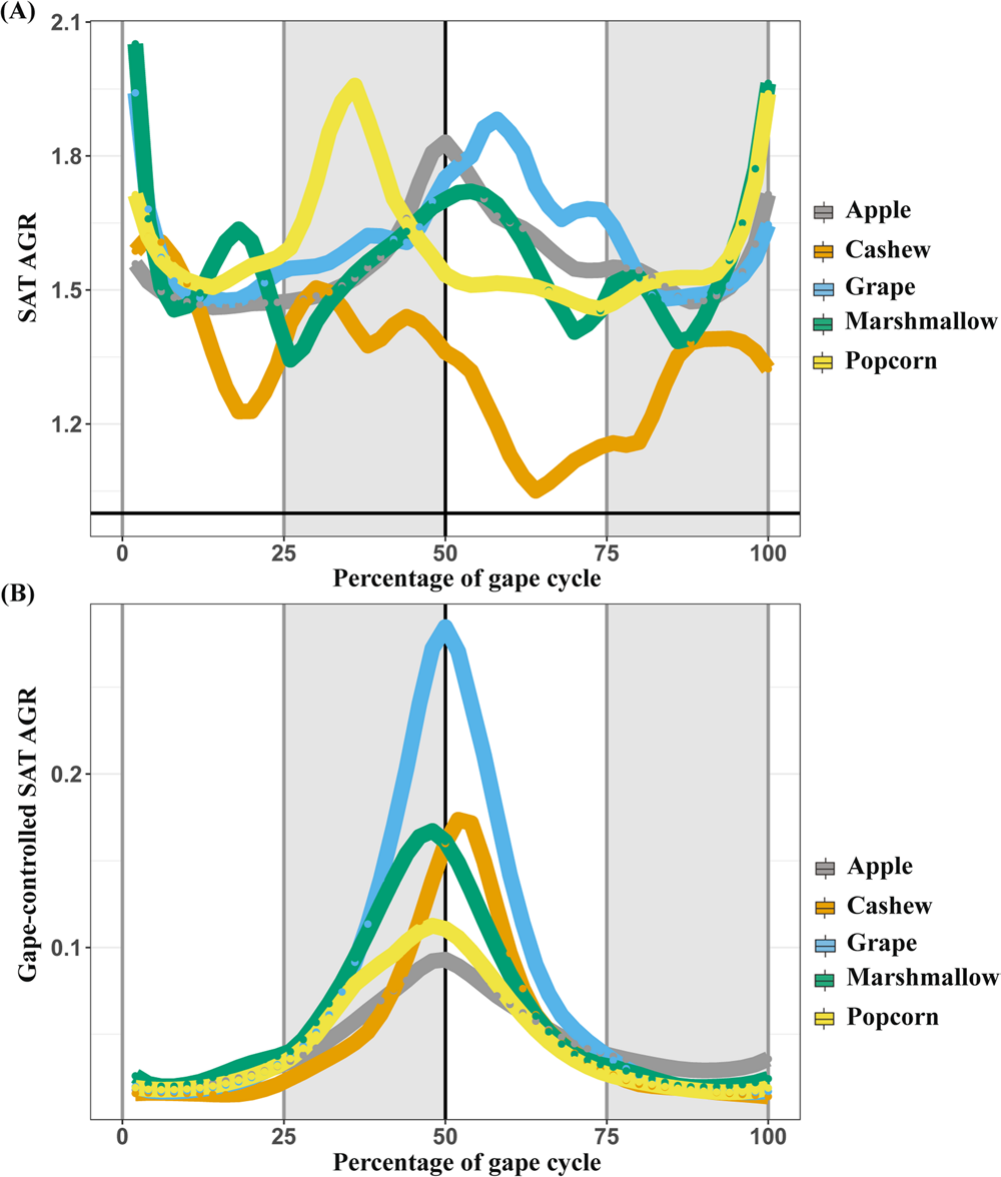
Architectural gear ratios (AGR) varied across the gape cycle. Across the entire gape cycle **(A)** AGR values decreased during jaw closing until the FC/SC transition and increased throughout SC. During jaw opening, AGR values decreased until the SO/FO transition and increased during FO. Across the entire gape cycle, gape-controlled AGR **(B)** had a single peak at the SC/SO transition. Figure generated in R (2017; https://www.R-project.org).

The SAT AGR varied across the gape cycle (Figs. 4C and 5A). From maximum gape, the SAT AGRs decreased during jaw closing, reaching their minimum values around 25% of the gape cycle. The SAT AGRs then increased slightly as the jaw elevated and the muscle shortened until around minimum gape (50% of the gape cycle). After minimum gape, as the jaw depressed slowly and the muscle slowly lengthened, the AGR decreased until approximately 75% of the gape cycle. The SAT AGRs then increased in the final 25% of the gape cycle as the jaw depressed more rapidly to maximum gape.

### Hypothesis 2: Fascicle velocity, muscle velocity and AGR vary across gape-cycle phases before and after controlling for gape

Fascicle velocity, muscle velocity and SAT AGR did not vary significantly between gape-cycle phases. Peak fascicle and muscle velocities and minimum SAT AGR values during jaw closing were synchronous with the FC/SC transition (*p* = 0.56; Fig. 5A). There were no significant differences in the timing of the FC/SC transition and minimum SAT AGR values during jaw closing between FMP groups. Minimum fascicle and muscle velocities and maximum SAT AGR were all synchronous with minimum gape and the SC/SO transition. There were no significant differences in the timing of the SC/SO transition and maximum SAT AGR values between FMP groups. Peak muscle velocity during jaw opening occurred at the same time as the SO/FO transition (*p* = 0.14); however, peak fiber velocity during jaw opening occurred significantly later than the SO/FO transition by 0.02 seconds or 5.2% (± 3.16% standard error of the mean) of the gape cycle (*p* = 0.04). The timing of minimum SAT AGR values during jaw opening was not significantly different (*p* = 0.63) from the SO/FO transition for all foods or for mechanically-challenging foods. However, the SO/FO transition occurred before minimum SAT AGR values during jaw opening for foods with lower FMPs (*p* = 0.03).

As predicted, after controlling for gape distance, fascicle and muscle velocities and AGRs during SC and SO were significantly higher than during FC and FO (all *p* < 0.01). After controlling for gape, fascicle and muscle velocities each had a single peak that occurred at approximately 22% of the gape cycle, and these peaks were not significantly different from the FC/SC transition. There were no significant differences (*p* = 0.49) in the timing of gape-controlled maximum fascicle or muscle velocity and the FC/SC transition between FMP groups. After controlling for gape, the SAT AGRs had a single peak 50% of the way through the gape cycle that was synchronous with the SC/SO transition (minimum gape) (Fig. 5B). There were no significant differences (*p* = 0.78) in gape-controlled AGR and the SC/SO transition between food groups.

### Hypothesis 3: SAT AGR varies with FMPs

**Figure 6:**
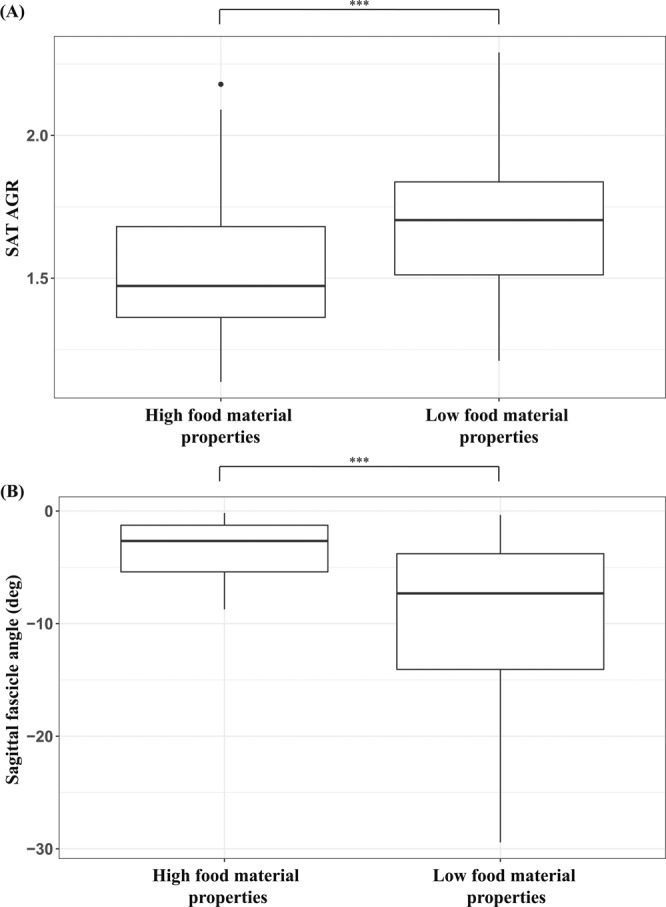
(**A**) Superficial anterior temporalis architectural gear ratios (SAT AGR) were lower during the gape cycle, and **(B)** sagittal pinnation angles were smaller, when subject animals fed on more mechanically challenging food items. The upper and lower bounds of the boxes correspond with the 25th and 75th percentiles and the whiskers extend 1.5 times the interquartile range in either direction. The median is represented by a horizontal line inside the boxes. A significance level, *p* < 0.01, is indicated by three asterisks. Figure generated in R (2017; https://www.R-project.org).

As hypothesized, across the gape cycle, the mechanically challenging foods—popcorn seeds and cashews—were associated with significantly lower AGRs (*p* < 0.01) (Fig. 6A). After controlling for gape, AGRs across the gape cycle were significantly lower for mechanically challenging foods (*p* = 0.03). Within gape cycle phases, only the SO phase had significantly lower AGR values for mechanically challenging foods before and after controlling for gape (both *p* < 0.01).

Foods with higher toughness and elastic modulus were associated with significantly smaller sagittal pinnation angles compared to less mechanically challenging foods (*p* < 0.01) (Fig. 6B). Coronal pinnation angle did not differ between FMP groups (*p* = 0.24)

## Discussion

Collectively, our results indicate that SAT AGR varies across the phases of the gape cycle during chewing. These results also suggest that muscle gearing may play an important role in modulating muscle performance at submaximal activation during naturalistic behaviors. We acknowledge that the small number of subjects and low statistical power limit the generalizability of our results. Additional studies on other mammalian species are needed to corroborate our findings. Here we discuss the implications of these initial results in terms of the impact of gape on architectural dynamics and the impact of AGR on muscle performance.

We found that during active chewing, gape distance within a gape cycle was highly correlated with changes in fascicle and muscle lengths and fascicle angles. Indeed, our *in vivo* results indicate that gape accounted for over 80% of variation in fiber length. Our *in vivo* findings are consistent with previous *in vitro* studies in macaques, which have shown that even with incremental stretching of anterior temporalis fibers from occlusion (e.g., 10% increments), fiber lengths undergo an average increase in length of ~1–2 mm such that anterior temporalis fibers are stretched by as much as 75% from occlusion to maximum gape^36^. With this fiber stretching, and coincident decreases in pinnation angles, temporalis PCSAs decreased by as much as 37% (this decrease was even greater for the superficial masseter). Collectively, these *in vivo* and *in vitro* results suggest that gape distance is a key driver of change in muscle architectural variables and force-generation capacity within a gape cycle.

When pinnate-fibered muscles shorten, they must bulge^49^, and the fascicles rotate as they shorten, altering the pinnation angle and the ratio of muscle belly shortening or lengthening to that of the fascicle^50,51^, i.e., the AGR^24^. Low AGRs favor muscle force production over muscle shortening velocity. During jaw closing, SAT AGRs were smallest at the FC/SC transition, suggesting that fascicles were shortening rapidly relative to other parts of the gape cycle. This shortening in the SAT was made possible by fascicle rotation, principally in the anteroposterior plane, and (presumably) by lengthening of series elastic elements in the muscle^52^. However, the influence of AGR on force production is dependent on pinnation angle, with larger pinnation angles resulting in lower force output. Coronal and sagittal fascicle angles were relatively low at the FC/SC transition and increased throughout the SC. Although we did not directly measure bite force, bite forces are first applied to a food item at the FC/SC transition^27^, suggesting that decreased AGRs and coronal and sagittal fascicle angles during jaw closing are likely associated with bite-force production. Gape-controlled maximum fascicle and muscle velocity also occurred at the FC/SC transition, as jaw-elevation velocity was slowed by the teeth coming into contact with the food, suggesting that changes in muscle architecture are not purely related to differences in gape. Maximum gape-controlled fascicle and muscle velocities at the FC/SC transition occurred as jaw elevation velocity is slowed by the teeth coming into contact with the food.

In contrast to jaw closing, fascicle and muscle velocities and AGRs are expected to show fewer differences during jaw opening^53^. Our results reveal that fascicle and muscle lengthening velocities increased during SO and decreased during FO. Fascicle and muscle velocities during jaw opening operated over the same dynamic range as the velocities during jaw closing (Fig. 3E,F). From maximum occlusion, SAT AGRs decreased to the SO/FO transition and then increased to maximum gape. We found that AGR values had approximately the same range of variation during jaw opening as during jaw closing. As the primate anterior temporalis is not active during jaw opening^47^, the temporalis undergoes passive changes in fascicle rotation while the muscle is lengthening. The presence of variable gearing during naturalistic contractions suggests that muscle architecture varies with both intra- and inter-cycle velocity and force changes. Future studies pairing electromyography with dynamic muscle architecture will address how muscle activation relates to the timing of jaw movements and fascicle and whole-muscle velocity.

We note that while fascicle and muscle lengths and fascicle angles are highly and significantly correlated with gape distance, these regressions are not isometric. The lack of isometry likely reflects a combination of factors including anatomical constraints and mandibular movements. For example, fascicles underwent twice as much rotation in the sagittal compared to coronal planes (Fig. 3C,D). These differences in magnitude of rotation potentially reflect anatomical constraints imposed on the SAT from the bony zygomatic arch, the thick fibrous temporal fascia overlying the temporalis muscle and the calvarial wall, all of which likely restrict the amount of bulging that the SAT can undergo in the coronal plane. Likewise, differences between the location of attachment of the temporalis muscle (the coronoid process) and the mandibular center of rotation in tufted capuchins (inferior to the mandibular condyle near the occlusal plane^8,54^) may also contribute to the variation between gape and muscle architecture variables. As a result of mandibular rotation, the coronoid process and attached internal tendon undergo over four times the displacement variation in the anteroposterior compared to the mediolateral plane during an average gape cycle. In this way, anatomical constraints and mandibular kinematics interact to drive variation in fascicle rotation.

Importantly, the SAT in primates and other mammals is only one region of a large muscle that is architecturally^54,55^ and physiologically^56^ complex and functionally heterogeneous (i.e., the SAT primarily elevates the jaw, whereas the posterior portions both elevate and retract the jaw^55^). We predict that dynamic muscle architecture of the temporalis will vary anteroposteriorly reflecting this functional heterogeneity. Other jaw elevators such as the superficial masseter may be relatively less anatomically constrained than the temporalis. Future studies examining muscle architectural variables in synergistic muscles will further clarify the influence of anatomical constraints and mandibular movements on architectural variables.

Architectural gear ratios varied with food material properties. Specifically, SAT AGRs (both before and after controlling for gape) were significantly lower for chews of popcorn seeds and cashews—more mechanically-challenging foods—and higher during gape cycles involving marshmallows, red grapes and apple pulp (Fig. 6A). Restricted fascicle rotation when chewing mechanically challenging foods limits muscle shape changes and is hypothesized to favor bite force production. We infer from these findings that dynamic changes in muscle architecture may influence muscle performance by potentially allowing the anterior temporalis to ameliorate force-gape constraints.

The magnitude of difference in the SAT AGRs between our “high” and “low” FMP groups was greater when gape was included in the analysis (i.e., when we did not control for gape). This finding suggests that gape may facilitate changes in the SAT AGRs, potentially to favor bite forces for mechanically challenging foods. Importantly, although SAT AGRs differed significantly between FMP groups, the toughness and elastic modulus values of the experimental foods used in this study are likely low compared to those of foods ingested and masticated by wild tufted capuchins^10^. We speculate that more mechanically challenging foods are likely to elicit even larger differences in AGR than those observed in this study. Additionally, the experimental foods used in this study exhibited some differences in volume (Supplementary Table 1) but did not elicit the relatively wide gapes that would require the SAT to stretch far beyond the optimal gape for force production in tufted capuchins. Tufted capuchin males can generate significantly wider maximum jaw gapes (both absolutely and relatively) compared with females (C. Vinyard, pers. comm), suggesting gape is likely to have a greater impact on architectural dynamics of the temporalis (and other jaw-elevator muscles) than observed in this study, which was restricted to females. Studies that include both males and females should further clarify the impact of gape variation on dynamic changes in muscle architecture.

The relationship between SAT AGRs and FMPs also likely varies across the chewing sequence. Previous studies^41–43,57–59^ suggest muscle activation, jaw kinematics, and bite forces vary across the chewing sequence, reflecting food breakdown and bolus formation. In particular, as a food is broken into particles, mixed with saliva, and formed into a bolus, it undergoes changes in size, shape, and bolus material properties^41,60^. However, XROMM recording limited our ability to capture the entire chewing sequence, resulting in our combining all gape cycles for analysis. Food geometry and FMPs are expected to have the greatest impact on the first few gape cycles in a chewing sequence^42^ and may account for some of the overlap in AGR in Fig. 6A.

Bite force estimates are key to understanding feeding-system design i.e., form-function relationships in mammals. Cadaveric specimens have been widely used to generate estimates of jaw-adductor muscle forces from architectural estimates of PCSAs^12,19,28–32^. These PCSA estimates have then been used to estimate bite forces for a variety of mammals^29,61–63^. While many estimates of jaw-elevator PCSAs have incorporated estimates of fiber pinnation angles^12,32,33^, these static pinnation angles do not capture the changes in pinnation that occur when fibers rotate during contraction. Our results empirically demonstrate changes in fascicle angles during active fiber rotation of the SAT and these fascicle angles in the sagittal plane are twice as large as fascicle angles in a coronal plane. Given that fibers rotate during muscle contraction, it is possible that including static pinnation angles in estimates of PCSAs could produce misleading results^13^. That said, despite the observed difference in magnitude of pinnation between the sagittal and coronal planes, the fiber rotations in these perpendicular planes had similar patterns of variation with gape such that the smallest fascicle angles occurred at minimum gape and the largest corresponded with maximum gape. These findings suggest that measuring pinnation angle in a standard plane, such as a coronal section, at a standard jaw posture, or with a gape correction, provides comparable pinnation angles despite *in vivo* differences in fiber rotation.

## Methods

### Subjects

Dynamic muscle architecture was quantified in the right SAT of three adult (ages 7–11 years) female *Sapajus apella* housed in the Animal Resources Center at the University of Chicago (UChicago) under approved Animal Care and Use Protocol (72430). During data recording, body masses ranged from 2.1 kg to 2.4 kg. All experiments were performed in accordance with the relevant guidelines and regulations.

### XROMM data collection and processing

Under general anesthesia, each animal was implanted with four small (1.0 mm) tantalum spheres (Bal-Tec, Los Angeles, CA) in the mandible, four in the cranium, and three in the SAT. The SAT markers had been laser drilled with a small diameter hole and pre-threaded with 6.0 Vicryl suture. At the point of greatest postorbital constriction, the anterior temporalis was divided parallel to the fascicles until the central myotendinous junction was exposed, and an individual fascicle could be traced from the central myotendinous junction to its superficial termination on the deep surface of the temporal fascia. Drilled tantalum spheres were sutured around either end of the selected fascicle, one at its central myotendinous junction and one at the superficial tendinous sheath (Fig. 2A). The third muscle marker was placed at the most superior attachment of the SAT, roughly in the same coronal plane as the fascicle markers. Fascicle markers were placed in homologous locations, but the exact location of the fascicles varied slightly between animals. Following marker implantation, the overlying temporal fascia was sutured closed to reduce the effect of aponeurosis modification on AGR^64^. All animals made a full recovery.

At least two weeks after marker placement, XROMM data were recorded in the University of Chicago XROMM Facility from each animal eating popcorn seeds, whole roasted cashews (without shells), half red seedless grapes, miniature marshmallows, and 1.5 cm diameter cubes of apple pulp (without skin). While popcorn seeds have markedly higher toughness and elastic modulus values compared to cashews, we considered these two foods to have ‘high’ FMP values compared to relatively compliant marshmallows, grapes, and apple pulp (Supplementary Table 1).

During feeding, the three-dimensional movements of all markers were recorded in a calibrated space using bi-planar videoradiography (90 kVp, 100 mA) at 150 frames per second (fps) using ProCapture software (Xcitex, Inc.). Subject-specific computerized tomography (CT) scans were made using a Vimago Robotic HDC scanner (Epica Medical Innovations), and the scans were processed in Horos (horosproject.org). Full procedures for surgically implanting radiopaque markers in bone, biplanar videoradiography, CT scanning and XROMM integration are thoroughly described elsewhere^65^. Precision tests for these techniques and markers indicate a marker spatial precision of 0.1–0.2 mm^66^.

Standardized grids were used to correct for X-ray image distortion during recording, and a cube with known geometry and positioning of radiopaque markers was used to calibrate the 3D space. XMALab (bitbucket.org/xromm/xmalab) was used to digitize the videoradiography data, and unless noted otherwise, all other analyses were performed in the open source software R^67^. The resulting XYZ coordinates of all markers were subjected to a low-pass Butterworth filter with 30 Hz cutoff frequency. The four cranial markers were fixed in space to correct for positional variation. Homologous transformation matrices were calculated for the mandibular markers from the rotation matrix and a translation vector using a singular value decomposition method. The processed CT scans were imported to Meshlab (meshlab.net) and landmarks were recorded at the right and left gonial angle and at the highest points on the coronoid processes. These landmark coordinates were multiplied by the homologous transformation matrix for each experiment to calculate their motion within the fixed cranial space.

Movements of the right anterior mandible marker were used to identify and separate the chewing sequences into individual gape cycles. Gape cycles were defined as sequential departure from, and return to, the point of maximum gape. One hundred and one gape cycles, 30–33 per animal, were included in these analyses (Supplementary Table 2). The number of recorded gape cycles varied by food type: apple (n = 13), cashew (13), grape (16), popcorn seed (16), and marshmallow (35). The material properties of these foods (Supplementary Table 1) are far below maximum values recorded in the lab (Laird, unpub. data) and reported for wild tufted capuchins^10^ making it reasonable to infer that these foods elicit submaximal muscle activation. At least two gape cycles were recorded for each animal per food type, except for popcorn seeds, which one animal would not eat. Between individuals, there were no significant differences in fascicle length or fascicle angles (*p* = 0.75) and chewing side did not significantly influence any of the results (*p* = 0.41), so all gapes were pooled. As a result of XROMM recording limitations (maximum recording duration of 10 seconds), recordings typically did not capture the full chewing sequence. Tufted capuchins showed minimal differences in gape-cycle duration between foods and across the chewing sequence^42^, so all gape cycles were combined in these analyses and standardized to 50 frames.

### Muscle architectural variables and gape cycle phases

Fascicle length was measured as the Euclidean distance between the markers at the central myotendinous junction and the superior attachment (Fig. 2B,C; Table 1); instantaneous fascicle velocity was the temporal derivative of this distance. Whole-muscle length was measured as the distance from the top of the coronoid process to the marker placed at the superior attachment of the SAT (Fig. 2B,C); instantaneous muscle velocity was the temporal derivative of this distance. Fascicle angle is used to describe angular differences associated with muscle fascicle rotation. These angles are not comparable to published static pinnation angles due to differences in measurement technique. Fascicle angles were calculated as the angle between the fascicle length and the distance from the central myotendinous junction marker to the superior attachment of the SAT. Sagittal fascicle angles were measured within sagittal planes and perpendicular to coronal planes; coronal fascicle angles were measured within coronal planes and perpendicular to sagittal planes (Fig. 1). Minimum or maximum values of all muscle architectural variables were scaled (normalized) to zero in order to compare measurements between animals. These normalized architectural variables then were averaged for each food type, and 25% smoothing span LOESS curves were fit to each food’s instantaneous fascicle and muscle velocities before calculating the AGRs. Averaged gape cycles combined gapes with slightly different jaw positions and timing, and smoothing was necessary to remove noise introduced during averaging. As a result of averaging, velocity LOESS curves did not reach zero at points of minimum and maximum gape for all food items. Muscle shape changes were calculated in both the anteroposterior and mediolateral planes as a maximum Euclidean distance between positions of the superficial fascicle marker within a gape cycle.

**Table 1:** Means (±standard deviations) of muscle architecture variables in the three tufted capuchins.

Dynamic muscle architecture	Sagittal fascicle angle (°)	Coronal fascicle angle (°)	Whole-muscle length (mm)	Fascicle length (mm)
Capuchin L	8.54 (± 2.76)	15.91(± 11.84)	89.36 (± 47.87)	9.01 (± 0.28)
Capuchin C	19.63 (± 1.72)	5.73 (± 1.45)	119.18 (± 23.86)	3.12 (± 0.80)
Capuchin A	28.54 (± 6.29)	32.96 (± 12.45)	91.17 (± 31.00)	4.03 (± 0.57)
Capuchin static muscle architecture	Pinnation angle 15.93 (8.01–23.81)		Fiber length 12.06 (10.4–14.93)	

Tufted capuchin static muscle architecture measurements are from Taylor and Vinyard (2009). Static pinnation angles are not comparable to fascicle angles due to different measurement protocols. Static fiber lengths are also notably longer compared with *in vivo* fascicle length measurements.

Gape distance is expected to have an important influence on dynamic changes in muscle architecture. Beyond the influence of gape, we expect muscle architecture to vary in association with changes in jaw acceleration and muscle activity, particularly bite force. Gape distance was measured as the Euclidean distance of mandibular displacement from the point of maximum occlusion and occurs primarily but not exclusively in a sagittal plane. Individual phases within a cycle were distinguished using instantaneous changes in jaw vertical acceleration, calculated using the second derivative of gape^42,59^. We also explored scaling relationships between gape distance and muscle architectural variables.

### Analyses

The experimental design of our study involves repeated measures e.g., multiple gape cycles while chewing a given food from each animal. We therefore tested each hypothesis using nested linear mixed-effect (LME) models fit by maximum likelihood because these models allow errors introduced by repeated measures to be dependent on each other. The structure of these models varied by hypothesis. In the first hypothesis, the explanatory variable was gape distance. Two sets of models were used to test the second hypothesis, with gape cycle phases and the timing of phase transitions as explanatory variables. Finally, FMP groups were explanatory variables for the third hypothesis. Random factors in each model were nested as gape cycle order within food type and food type within subject. This approach allows assessment of inter-individual differences on gape cycle order within a chewing sequence, differences within a gape cycle and variation between foods. The LME models were analyzed using ANOVAs and posthoc Tukey comparisons performed in the R package ‘multcomp’ and the sequential Bonferroni adjustment used to minimize type one error^68^. As a means of comparing the LME models and to determine how well each model fit the data, the R package MuMIn^69^ was used to calculate: the marginal R^2^ values (R^2^m), quantifying how well the fixed terms fit the model; and the conditional R^2^ values (R^2^c), quantifying the total model fit including random and fixed terms. Significance was set at *p* < 0.05. Reduced major axis regressions were used to address the scaling relationships between gape distance and muscle architecture variables.

## Supplementary information

Supplementary information.

## Data Availability

All raw data are available on the University of Chicago X-Ray Motion Analysis (XMA) research portal.
