# Supplementary material for: Muscle architecture dynamics modulate performance of the superficial anterior temporalis muscle during chewing in capuchins
Source: Sci Rep. 2020 Apr 14;10:6410. doi: 10.1038/s41598-020-63376-y (PMC7156371; doi:10.1038/s41598-020-63376-y)
Supplement: Supplementary file 1 — Supplementary information. [file 41598_2020_63376_MOESM1_ESM.docx]

**Muscle architecture dynamics modulate performance of the superficial anterior temporalis muscle during chewing in capuchins**

Myra F. Laird^1*^, Michael C. Granatosky^2^, Andrea B. Taylor^3^, Callum F. Ross^4^

^1^ Department of Integrative Anatomical Sciences, University of Southern California, Los Angeles, CA USA

^2^ Department of Anatomy, New York Institute of Technology, Old Westbury, NY USA

^3^ Basic Sciences Department, Touro University, Vallejo, CA USA

^4^ Department of Organismal Biology and Anatomy, University of Chicago, Chicago, IL USA

**Supplementary Information**

| **Food** | **Food volume (SD)** **mm^3^** | **Toughness (SD) Jm^-2^** | **Elastic Modulus (SD) MPa** | **FMP Group** |
| --- | --- | --- | --- | --- |
| Marshmallow | 1249.61 (± 47.06) | 2.08 (± 1.31) | 0.03 (± 0.01) | Low |
| Red Grape | 1507.03 (± 285.14) | NA | NA | Low |
| Apple pulp | 3565 (± 390.58) | 56.97 (± 17.76) | 3.41 (± 0.10) | Low |
| Cashew | 960.29 (± 426.42) | 174.8 (± 44.4) | 11.08 (± 2.28) | High |
| Popcorn seed | 100.86 (± 7.29) | 2978.82 (± 678.34) | 325.4 (± 218.83) | High |

**Supplementary Table 1.** Volume, toughness, and elastic modulus of foods included in this study. Toughness and elastic modulus values of apple pulp and popcorn seeds are from Williams et al. (2005); cashew measures are from Agrawal et al. (1997). Marshmallow toughness and elastic modulus measures were collected for this study. Apple and cashew volumes were calculated as cubes, marshmallow and half red grape volume was calculated as a cylinder, and popcorn seed volumes were calculated as spheres.

| **Food** | **Capuchin A** | **Capuchin C** | **Capuchin L** |
| --- | --- | --- | --- |
| Apple (without skin) | 3 | 3 | 6 |
| Cashew (without shell) | 6 | 3 | 4 |
| Grape | 2 | 14 | 8 |
| Marshmallow | 19 | 7 | 10 |
| Popcorn seeds | - | 4 | 12 |

**Supplementary Table 2.** Number of chewing cycles analyzed for each animal and food type. Capuchin A did not eat popcorn seeds.

**Supplementary References**

Williams, S. H., Wright, B. W., Truong, V. D., Daubert, C. R., & Vinyard, C. J. (2005).

Mechanical properties of foods used in experimental studies of primate masticatory function. American Journal of Primatology, 67(3), 329-346.

Agrawal, K. R., Lucas, P. W., Prinz, J. F., & Bruce, I. C. (1997). Mechanical properties of foods

responsible for resisting food breakdown in the human mouth. Archives of Oral Biology, 42(1), 1-9.
